# Crosstalk between tubular epithelial cells and glomerular endothelial cells in diabetic kidney disease

**DOI:** 10.1111/cpr.12763

**Published:** 2020-01-11

**Authors:** Si‐Jie Chen, Lin‐Li Lv, Bi‐Cheng Liu, Ri‐Ning Tang

**Affiliations:** ^1^ Institute of Nephrology Zhongda Hospital Nanjing Lishui People's Hospital Nanjing China; ^2^ Institute of Nephrology Zhongda Hospital School of Medicine Southeast University Nanjing China

**Keywords:** crosstalk, diabetic kidney disease, glomerular endothelial cells, tubular epithelial cells

## Abstract

In recent years, although the development of clinical therapy for diabetic kidney disease (DKD) has made great progress, the progression of DKD still cannot be controlled. Therefore, further study of the pathogenesis of DKD and improvements in DKD treatment are crucial for prognosis. Traditional studies have shown that podocyte injury plays an important role in this process. Recently, it has been found that glomerulotubular balance and tubuloglomerular feedback (TGF) may be involved in the progression of DKD. Glomerulotubular balance is the specific gravity absorption of the glomerular ultrafiltrate by the proximal tubules, which absorbs only 65% to 70% of the ultrafiltrate. This ensures that the urine volume will not change much regardless of whether the glomerular filtration rate (GFR) increases or decreases. TGF is one of the significant mechanisms of renal blood flow and self‐regulation of GFR, but how they participate in the development of DKD in the pathological state and the specific mechanism is not clear. Injury to tubular epithelial cells (TECs) is the key link in DKD. Additionally, injury to glomerular endothelial cells (GECs) plays a key role in the early occurrence and development of DKD. However, TECs and GECs are close to each other in anatomical position and can crosstalk with each other, which may affect the development of DKD. Therefore, the purpose of this review was to summarize the current knowledge on the crosstalk between TECs and GECs in the pathogenesis of DKD and to highlight specific clinical and potential therapeutic strategies.

## 1 INTRODUCTION

Diabetic kidney disease (DKD) is the primary cause of end‐stage renal disease (ESRD), and it has become a public issue that seriously endangers human health. It is necessary to explore the pathogenesis of DKD and find effective treatments to improve the prognosis of DKD.

DKD is characterized by glomerulosclerosis, tubulointerstitial fibrosis and renal vascular disease.^1^ Glomerular endothelial cells (GECs) are one of the inherent cells of the glomerulus. As the first barrier of the glomerular filtration membrane, GECs are in direct contact with circulating substances in the blood and are more likely to be damaged by glucose, lipids and inflammatory factors. GECs play an important role in the occurrence and development of DKD.^2^, ^3^ The renal tubule interstitium accounts for more than 90% of renal parenchyma and performs a variety of functions. The surface of the renal tubule cavity is accompanied by a brush edge, which can increase the cell surface area and facilitate reabsorption in the renal tubule. In addition, GECs are metabolic cells that are rich in mitochondria, lysosomes and other organelles; require a large amount of energy; and are sensitive to damage.^4^ In the diabetic environment, tubular epithelial cells (TECs) are easily affected by metabolic disorders, inflammatory states and changes in urine composition and haemodynamics, resulting in oxidative stress and secretion of a variety of cytokines, leading to interstitial inflammation and fibrosis.

**Figure 1:**
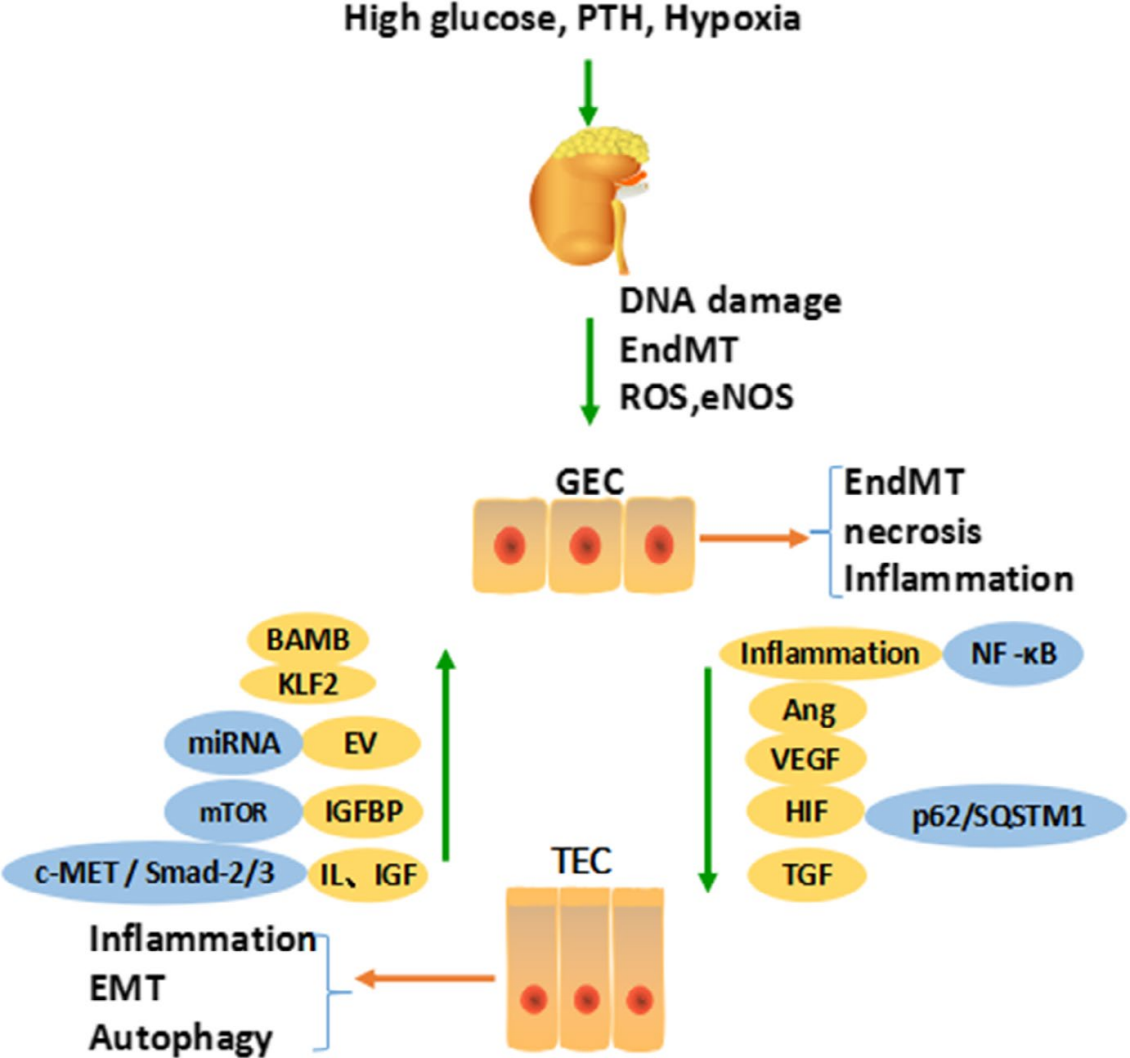
There are many common signalling pathways between TECs and GECs, in which crosstalk plays a vast role. During the occurrence of DKD, abnormal secretion of VEGF, Ang‐1, inflammatory factors and hypoxia promote injury to GECs. Moreover, injured GECs secreting HGF, IGFBPs, EVs, KLF and autophagy can also act on TECs, causing changes in the structure and function of TECs to different degrees. Parathyroid hormone (PTH), glomerular endothelial cell (GEC), tubular epithelial cell (TEC), endothelial‐mesenchymal transition (EndMT), epithelial‐mesenchymal transition (EMT), reactive oxygen species (ROS), endothelial nitric oxide synthase (eNOS), angiopoietin (Ang), vascular endothelial growth factor (VEGF), hypoxia‐inducible factor (HIF), tubuloglomerular feedback (TGF), Kruppel‐like factor (KLF), hepatocyte growth factor (HGF/c‐MET), bone activin membrane binding (BAMB), extracellular vesicles (EV), insulin‐like growth factor binding proteins (IGFBPs), insulin‐like growth factor (IGF)

Recent studies have shown that glomerulotubular balance and tubuloglomerular feedback (TGF) affect the progression of DKD. Based on this, clinical application of novel hypoglycaemic drugs such as daglitazine, a sodium‐glucose cotransporter‐2 (SGLT2) inhibitor, can increase urinary glucose excretion, control blood sugar and reduce glomerular injury, thereby delaying the deterioration of renal function in DKD. Nektaria found that there are many common signalling pathways between TECs and GECs, in which crosstalk plays a vast role (Figure 1).^5^ During the occurrence of DKD, abnormal secretion of vascular endothelial growth factor (VEGF), angiopoietin‐1 (Ang‐1) and inflammatory factors and hypoxia promotes injury to GECs. Moreover, injured GECs secrete hepatocyte growth factor (HGF), insulin‐like growth factor binding proteins (IGFBPs), extracellular vesicles (EVs) and Kruppel‐like factor (KLF), and autophagy can also act on TECs, causing changes in the structure and function of TECs to different degrees. Therefore, the purpose of this review was to briefly summarize the current knowledge on the pathogenesis of DKD, with specific comments on the crosstalk between GECs and TECs and potential therapeutic interventions.

## 2 TEC INJURY AND DKD

Compared with GEC lesions, TECs are more closely related to the deterioration of renal function.^6^ There are different degrees of renal tubular injury in the early stage of DKD. It has been reported that only 1% of diabetic microalbuminuria patients have typical glomerular structural damage, while 1/3 of patients have no or very slight glomerular injury,^7^ but renal tubular injury is serious. The manifestations are thickening of the renal tubular basement membrane, tubular inflammatory lesions, renal tubular atrophy, increased apoptosis, interstitial fibrosis and thinning of peritubular capillaries.^8^

It has been found that massive proteinuria in DKD patients causes inflammatory responses, oxidative stress, activation of transforming growth factor‐β (TGF‐β) and the renin‐angiotensin system (RAS) and accumulation of advanced glycation end products (AGEs),^9^ resulting in changes in TEC function and morphology, epithelial cell hypertrophy, epithelial‐mesenchymal transition (EMT), epithelial cell detachment and apoptosis. Exfoliation and apoptosis of TECs eventually lead to renal fibrosis and promote the progression of DKD to ESRD.^10^ Existing studies have shown that DKD research should not focus on changes in epithelial cell structure or specific proteins. More attention should be paid to the related factors affecting the gene expression of epithelial cells and the interactions between other cells (macrophages, etc) and TECs.^11^

## 3 GEC INJURY AND DKD

GECs are a fundamental part of the renal filtration barrier, which is in direct contact with the blood circulation and is easily affected or damaged by circulating substances such as blood glucose, lipids and inflammatory factors. In the case of high glucose, metabolites and other stimuli activate a variety of signalling pathways (such as RAS, AGEs, polyol pathway and the protein kinase C pathway) and induce intrinsic renal cells to produce a variety of growth factors, cytokines, reactive oxygen species (ROS) and endothelial nitric oxide synthase (eNOS), in addition to mitochondrial DNA damage, and inflammatory responses, resulting in GEC dysfunction.^12^ Our previous studies found that high glucose mediated IL to induce the endothelial‐mesenchymal transition (EndMT) of GECs.^13^ Damaged GECs can affect haemodynamic changes, damage the filtration membrane charge barrier and change permeability, which is closely related to the production of proteinuria, thus promoting the occurrence and development of DKD. With the loss of extracellular sugar calyx, increase in ROS production, EndMT changes and many other factors, renal balloon pressure increased and accelerated the process of renal fibrosis. The use of the plant water–soluble phenolic acid salvianolic acid A (SalA) reduces the actin cytoskeleton rearrangement induced by AGEs and restores the permeability of GECs by inhibiting the AGE‐RAG‐RhoMROCK pathway. In addition, SalA alleviates AGE‐induced oxidative stress and renal structural damage through the AGE‐RAGE‐Nox4 axis. Thus, SalA can effectively improve the early DKD.^14^

In recent years, many studies have confirmed that under the influence of high glucose, endothelial cells lose their markers and cellular adhesions through the TGF‐β/Smad‐dependent signal transduction pathway, obtain markers of mesenchymal cells and gradually differentiate into mesenchymal cells. EndMT is one of the mechanisms of renal fibrosis in DKD. Li found that EndMT occurred in approximately 30% of DKD cases. The classical signal Wnt is produced in the myocardial infarction in mouse model, and it has been confirmed that classical Wnt signalling induces the EndMT. Most mesenchymal cells derived from the transdifferentiation of endothelial cells are myofibroblasts (MyoFbs).^15^ MyoFbs are the main source of mesenchymal cells that secrete extracellular matrix and play a major role in promoting early renal fibrosis in DKD. If MyoFbs persist, they cause excessive accumulation of extracellular matrix, glomerulosclerosis and tubulointerstitial fibrosis, destroying the normal structure and function of the kidney and eventually leading to ESRD. Therefore, paying attention to the protection of GECs in early DKD is of great significance in delaying the progression of DKD and improving the prognosis of DKD.^16^

## 4 THE ROLE OF CROSSTALK BETWEEN TECS AND GECS IN DKD

Because of the relationship between the location of TECs and GECs, their abnormal crosstalk plays a key role in the pathogenesis of DKD.

Alterations in the GEC surface layer, including its major component glycocalyx, are a leading cause of microalbuminuria observed in early DKD. In addition, recent studies suggest that GECs contribute to DKD by paracrine communication with other glomerular cells, such as TECs.^17^ Emerging evidence suggests that the glomerular filtration barrier and tubulointerstitial compartment are a composite, dynamic entity where any injury of one cell type spreads to other cell types and leads to the dysfunction of the whole apparatus. Gene and protein expression profiling show that glomerular changes in diabetes involve many metabolic and signalling pathways that may occur in individual glomerular cells or through crosstalk between them.^18^

In DKD, TECs induce cascading inflammatory responses by releasing cytokines, miRNAs and extracellular vesicles through autocrine or paracrine mechanisms under conditions of high glucose and proteinuria.^19^ The glomerular vascular network undergoes apoptosis, necrosis and transdifferentiation with the stimulation of inflammatory factors, and the structure and function of GECs are destroyed. Damaged GECs reduce the blood supply to the renal tubules, leading to exacerbated TEC damage. In the future, more efforts need to be made to build a kidney cell map and understand the crosstalk between cells.^20^

### 4.1 TEC damage caused abnormalities in GECs

#### 4.1.1 The inflammatory response of TECs contributes to the injury of GECs

DKD is considered to be an inflammatory disease caused by disorders of glucose and lipid metabolism.^21^ Our previous study found that proteinuria causes inflammation of TECs in a CKD mouse model.^22^ High concentrations of urinary albumin in DKD patients activate TECs to produce proinflammatory factors such as CRP, IL, TNF‐α, NF‐κB and ROS, which can lead to GEC injury, apoptosis and EndMT. Inflammation leads to lipid peroxidation, membrane structure destruction, membrane protein function inhibition and membrane transport system dysfunction, such as sodium pump, calcium pump and G protein–coupled receptor dysfunction, which induces GEC apoptosis. In our in vitro experiment, we found that IL‐1β activates the EndMT induced by high glucose in endothelial cells.^13^ ROS lead to dysfunction of GECs through activation of PKC‐dependent NADPH oxidase. Several subtypes of PKC have been shown to be involved in the regulation of GEC dysfunction in DKD, in which activation of PKC‐β promotes proteinuria. In the diabetic mouse model with specific knockout of PKC‐β, proteinuria was less than that in the control group or in the DKD mice in which proteinuria was alleviated.^23^

DPP‐4 inhibitors act on distal convoluted tubules, reducing oxidative stress in renal tissue, preventing GEC damage caused by inflammatory factors and reducing urinary albumin excretion. In animal experiments, siglitine, liglitine and verglitine were observed to directly reduce the excretion of urinary albumin. Clinical studies have shown that siglitine can reduce the excretion of urinary albumin in DKD patients. Siglitine reduces urinary microalbuminuria, and riglitine reduces the urinary albumin/creatinine ratio.^21^ TEC‐induced inflammation induces GEC damage, apoptosis and EndMT through NF‐κB and other signalling pathways.

#### 4.1.2 Angiopoietin‐1/Angiopoietin‐2 (Ang‐1/Ang‐2)‐Tie2–mediated crosstalk between TECs and GECs in DKD

Ang‐1 and Ang‐2 are essential for the formation of mature blood vessels and act by binding to endothelial‐specific receptor tyrosine kinase‐2 (Tie‐2). Their functions are different. High glucose activates RAS in the kidney. TECs produce Ang‐1, which binds to the Tie‐2 site of GECs, reducing the permeability of GECs and regulating the effect of VEGF to maintain GEC homeostasis. Ang‐2 is generated by GECs and competitively inhibits the binding of Ang‐1 to Tie‐2.^24^ When DKD occurs, it is accompanied by a decrease in the proportion of Ang‐1/Ang‐2. Rizkalla et al reported that after 8‐week‐old adult rats were given STZ, Ang‐1 was lower than that of non‐diabetic controls, while Ang‐2 increased. Furthermore, Jeansson et al compared STZ‐induced diabetic mice with Ang‐1 knockout mice. Ang‐1 gene knockout accelerated diabetes‐mediated glomerular injury, suggesting that Ang‐1 may protect GECs. Overexpression of Ang‐1 in TECs alleviates albuminuria and prevents the progression of DKD by increasing Tie‐2 phosphorylation. Our study found that Ang‐2 blockers slow the occurrence and development of DKD.^25^

As an anti‐aging gene, Klotho encodes a wide range of biological effects, playing a protective role in a variety of acute and chronic kidney diseases and is closely related to the occurrence and development of DKD. In the kidney, Ang‐2 inhibits the expression of Klotho, and Klotho in the kidney, which has high affinity through the Ang‐1 receptor, decreases the FGF23‐K1 receptor complex, which leads to FGF23 resistance. Thus, activation of vitamin D is inhibited, and GEC function is damaged. Recently, it has been reported that Klotho can directly bind to VEGFR and instantaneous receptor potential channel 1 (TPRC‐1) to enhance the interaction between the two, thus regulating the influx of Ca^2+^ to maintain vascular endothelial biological homeostasis.^26^ In contrast, Klotho deficiency leads to continuous enhancement of Ca^2+^ influx and excessive activation of Ca^2+^‐dependent protease, resulting in GEC damage and increased vascular permeability. In the rat model of renal proteinuria induced by puromycin aminonucleoside, the expression of Klotho decreased while the level of proteinuria increased. In DKD models, RAAS blockers can reduce the expression of FGF‐23 in the whole body and kidney, increasing the expression of Klotho at the same time. The Ang‐2 produced by TECs regulates the steady state and function of GECs through Tie‐2 and VEGF.

#### 4.1.3 The vascular endothelial growth factor/vascular endothelial growth factor receptor (VEGF/VEGFR) axis–induced response in TECs promotes injury to GECs

VEGF, secreted by TECs, regulates the structure and function of GECs by binding to the corresponding VEGFR expressed by GECs, forming the VEGF/VEGFR axis. The VEGF/VEGFR system is very important for normal glomerular development and adult renal dynamic balance, and changes in this system play a major role in the pathophysiological progression of DKD.^27^ Increased expression of VEGF/VEGFR in early DKD leads to neovascularization and other glomerular injuries. VEGF‐A is the most useful subtype of VEGF. With the exacerbation of TEC damage and deletion in the late stage of DKD, the production of VEGF‐A decreases, reducing VEGF‐A‐mediated protection of the structure and function of GECs and promoting GEC damage. When TEC expression of VEGF‐A in diabetic mice is specifically knocked out or upregulated, GECs could not maintain normal function, thus accelerating GEC damage. eNOS deficiency caused by GEC damage, combined with the effect of hyperglycaemia, leads to TEC damage, and damaged TECs cannot produce enough VEGF‐A, leading to a vicious cycle.^28^ Expression of VEGF is decreased in renal biopsy tissues of diabetic patients, and expression of VEGF is negatively correlated with proteinuria, while patients with VEGF antibodies were at risk of GEC injury. The VEGF/VEGFR axis plays an important role in TEC sand GECs, which leads to a vicious cycle in DKD.

#### 4.1.4 Hypoxia‐induced responses in TECs promote injury to GECs

High glucose can cause hypoxia. Chronic hypoxia is associated with the occurrence and development of DKD. Expression of hypoxia inducible factor‐1α (HIF‐1α) in TECs is increased, and HIF‐1α enters the nucleus and binds with the HIF‐1β subunit at the hypoxia response element (HRE) to form the dimer complex HIF‐1, which activates the downstream inflammatory response to promote GEC proliferation and EndMT, inducing autophagy and programmed cell death.^29^ Hypoxia increases the expression of Runx1 in GECs. Under hypoxia, transcription of the inflammatory molecules IL‐1β, ICAM and TNF‐α increases. The stability of the cell membrane decreases, and the apoptosis pathway is activated. Inhibiting Runx1 significantly decreases the hypoxia‐induced expression of the inflammatory molecules IL‐1β, ICAM and TNF‐α, and injury to the GEC membrane and the apoptosis pathway are inhibited.

High mobility group box‐1 (HMGB1), a member of the HMG‐box family, is a highly rich and widely expressed protein that is highly expressed by TECs and stimulates inflammatory signalling pathways such as the inflammasome and NF‐kB pathways under anoxic conditions. HMGB1 induces the release of proinflammatory cytokines and affects the function of GECs.^30^ These results suggest that hypoxia leads to GEC damage in a manner that is dependent on TEC secretion of HIF‐1 and the HMGB1 pathway.

#### 4.1.5 TGF‐mediated crosstalk between TECs and GECs in DKD

TGF is a main mechanism for regulating renal microcirculation and renal haemodynamics. Macula densa (MD), which is composed of TECs, regulates the contraction and relaxation of glomerular arterioles and changes the GFR. SGLT2 expressed by proximal TECs determines the reabsorption of renal blood glucose.^31^ In DKD patients, more glucose molecules enter the renal tubules, which results in increased SGLT2 expression, a large number of Na+ and glucose molecules transporting back to the blood through SGLT2, and decreased Na+ flowing through the MD. In this condition, TGF is weakened by releasing nitric oxide (NO), RAS and so on.^32^ The glomerular arterioles become dilated, and the GFR increases. SGLT‐2 inhibitors can directly reduce high glomerular perfusion, high pressure and high filtration and inhibit the expression of inflammatory factors and the GEC fibrosis by promoting urinary sodium excretion and repairing TGF.^33^ TGF is regulated by many factors, including Ang‐2, adenosine, arachidonic acid metabolite, ATP, atrial natriuretic factor and NO. The team led by Schnermann believes that MD cells consume ATP to produce metabolites, especially adenosine, during NaCl transport, entering the proximal space through the basolateral membrane and coupling with different adenosine receptor subtypes expressed in GECs or interstitial cell membranes.^30^ The proliferation and migration of GECs are induced by the upregulation of angiogenic factors such as VEGF, IGF‐1, FGF, IL‐8 and Ang‐2 and the downregulation of angiogenic factors such as TNF‐ɑ. In addition, ATP plays a key role in TGF. Pyroptosis is a programmed cell death mode related to inflammation, which is mediated by an aspartic acid proteolytic enzyme (caspase‐1) containing cysteine, accompanied by the release of a large number of proinflammatory factors, inducing cascade amplification of the inflammatory response.^34^ TECs can change the structure and function of GECs through SGLT2, adenosine, ATP and RAAS, which are activated by TGF.

### 4.2 GEC injury causes abnormalities in TECs

#### 4.2.1 Kruppel‐like factor (KLF)–mediated EMT in GECs promotes injury to TECs

KLF is a shear stress‐induced transcription factor that has endothelial protective effects.^35^ Glomerular injury and proteinuria in GEC‐specific KLF2 heterozygous knockout diabetic mice were significantly worse than those of wild‐type diabetic mice. The low expression of KLF2 in GECs differentially regulates the expression of key angiogenic markers. Moreover, the decrease in endothelial‐specific KLF2 also leads to the obvious injury of TECs in diabetic mice, indicating that KLF2 may be involved in GEC/TEC interactions.^36^ Studies have shown that KLFs negatively regulate the proliferation of TECs by inducing G1 arrest in TECs.^37^ In addition, it has been found that Dnmt1 is involved in the TGF‐β1–mediated methylation of the KLF4 promoter in vivo and in vitro. Overexpression of EMT in TECs and overexpression of KLF4 induce the expression of E‐cadherin and ZO‐1, which downregulates the expression of α‐SMA and fibroblast specific protein‐l (FSP‐1), thus inhibiting the process of EMT. KLF6 is expressed in proximal TECs. KLF6 can transcriptionally regulate the expression of TGF‐β and affect the process of EMT.^38^ As a result, KLF6 mediates injury to TECs by GECs.

#### 4.2.2 Hepatocyte growth factor (HGF/c‐MET) inhibits EMT in TECs by affecting GECs

HGF is a pluripotent factor found in mature hepatocytes that can promote cell proliferation, division, differentiation and migration.^39^ HGF is mainly produced by GECs and mesangial cells in the kidney, and TECs express its receptor c‐MET. The expression of HGF in serum and renal tissue stimulates angiogenesis, induces renal tubule formation and alleviates renal interstitial fibrosis, glomerulosclerosis and renal tubular cystic degeneration. High glucose stimulates the expression of cytokines such as IL‐6 and insulin‐like growth factor (IGF) and upregulates the expression of c‐MET in TECs, inducing TGF to inhibit the expression of HGF. This kind of negative feedback regulation mechanism plays a significant role in renal interstitial fibrosis and sclerosis in DKD. However, a low dose of HGF inhibits apoptosis of GECs and protects cytoskeletal proteins. Although the specific mechanism of HGF is not clear, its relationship with c‐MET is a classical way to study the effect of GECs on TECs.^40^

HGF inhibits the transdifferentiation of TECs. The EMT involves remodelling the transcription and regulation of the cytoskeleton, adhesion between cells and matrix binding between cells. This eventually leads to the transdifferentiation of TECs into fibroblasts, which increases the mobility and invasive ability of these cells. HGF can inhibit the EMT by blocking the Smad‐2/3 signalling pathway. Li et al cultured human TECs in a high glucose environment in vitro and found that the regulation of SnoN protein occurs mainly through the TGF‐β/Smad signalling pathway to increase the expression of Smad ubiquitin regulatory factor 2 (Smurf2). HGF inhibits the ubiquitin‐proteasome–dependent degradation of SnoN protein by downregulating the expression of Smurf2 and antagonizing the development of the EMT.^39^ GECs secrete HGF to protect TECs from the EMT.

#### 4.2.3 Insulin‐like growth factor binding proteins (IGFBPs) mediate crosstalk between TECs and GECs in DKD

IGFs are a group of peptides with a growth‐promoting effect. IGF‐1 mRNA and protein are distributed in GECs. Imbalance in the IGF system occurs in the early stage of DKD and is related to glomerular hypertrophy and microalbuminuria. IGF‐1 and IGF‐2 are structurally similar to insulin and can convey signals through the insulin/IGF receptor family. Studies have shown that TECs are specific targets for insulin or IGF, and epithelial cell‐specific insulin signal transduction is biologically important for normal glomerular function and DKD.^41^ IGFBPs can enhance or inhibit the biological effect of IGF by regulating the binding of IGF to its specific receptor. GECs produce IGFBP‐4, IGFBP‐2 and IGFBP‐3 and express mRNA for IGFBP‐2 to IGFBP‐5, which regulate IGF signal transduction in TECs. Inhibition of the IGF signalling pathway can inhibit TECS6 ribosomal protein hyperphosphorylation and block activation of the mTOR pathway without affecting mitochondrial structural defects, reducing kidney disease and providing a new target for the treatment of DKD. IGFBPs can mediate crosstalk between TECs and GECs in DKD.

#### 4.2.4 Autophagy maintains balance between GECs and TECs

Autophagy is an important mechanism for maintaining environmental stability in glomeruli and renal tubules, which is necessary in human health and diseases. Bone morphogenetic protein (BMP) and activin receptor membrane binding inhibitor (BAMBI), a competitive receptor antagonist of the gfb receptor family, are expressed in GECs and regulated by autophagy in TECs.^42^ High glucose inhibits autophagy and results in p62/SQSTM1 protein accumulation in the TECs of DKD patients, while p62/SQSTMl is mainly degraded by autophagic lysosomes, suggesting that the autophagy pathway is inhibited in renal tissue. Autophagy enlarges and deforms TECs and gradually results in deformed mitochondria. p62 and ubiquitin are positive factors that lead to apoptosis of TECs. SGLT2 inhibitors increase urinary glucose excretion and lower blood glucose levels in the kidney. Knockout of SGLT2 attenuates the accumulation of p62/SQSTM1 induced by STZ, indicating that glucose uptake induced by SGLT2 can inhibit autophagy.

In addition, autophagy induced by GECs can reduce tubulointerstitial fibrosis. This antifibrotic effect may be closely related to the cell cycle. Inhibition of autophagy can induce cells to stay in the G2 phase, increasing extracellular matrix deposition, and exacerbating TECs in the G2 phase in renal fibrosis injury. The increase in the proportion of G2 phase cells has been proven to be closely related to the deposition of fibrin in renal tissue and the injury of renal tissue.^43^ GECs induce structural and functional changes in TECs through autophagy. In vivo experiments showed that specific knockout of the autophagy protein 5 (Atg5) gene, an important component of autophagy, leads to increased proteinuria and upregulation of oxidative stress in older rats, resulting in glomerulosclerosis, TEC damage and apoptosis. These results again emphasize the important role of GEC‐induced autophagy in the development of DKD.

#### 4.2.5 Extracellular vesicles (EVs) mediate crosstalk between TECs and GECs in DKD

EVs are spherical membranous vesicles surrounded by lipid bilayers. The contents of EVs include proteins, lipids, nucleic acids and membrane receptors from parental cells. EVs are released into the extracellular space to enter body fluids and reach distant tissues. GECs secrete EVs containing TGF‐β, miRNA and other molecules to participate in EMT, activating the smad3 signalling pathway in TECs. High glucose–stimulated GECs secrete EVs that contain TGF‐β and miRNA. When taken up by TECs, EVs release TGF‐β and miRNA. The APl transcription factor complex binds to the promoter and participates in the phenotypic changes of EMT by activating the smad3 signalling pathway, which affects the permeability of the filtration membrane and exacerbates the DKD process.^44^ TECs also secrete EVs that target NF‐κB, which activates the inflammatory response and induces GEC dysfunction. In addition, studies have shown that external injuries such as hypoxia, proteinuria or physical damage trigger the release of EVs from TECs carrying specific cargo. HIF‐1 promotes the production of exocrine bodies in TECs under hypoxia. EVs from TECs may be docked on GECs, promoting endothelial dysfunction or repair.^45^ Wang X et al studied the difference in the expression of exocrine miRNA and protein in human primary renal tubular epithelial cells (PTECs) under normal, inflammatory and anoxic conditions. The expression of the exocrine marker proteins CD9, CD63 and CD81 was increased. High glucose induces the upregulation of miR‐27a, miR‐155, miR‐19b‐3p and miRNA‐23a expression in TECs and activation of the inflammatory response by targeting NF‐κB.^46^ Inflammatory factors act on vascular endothelial cells, inducing expression of procoagulant factors and adhesion molecules that adhere to inflammatory cells, promoting intravascular thrombosis, increasing capillary permeability and inducing endothelial cell dysfunction.

#### 4.2.6 Glomerulotubular balance maintains balance between GECs and TECs

The glomerulotubular balance is a phenomenon in which the reabsorption of water and solutes by the proximal tubules changes with the GFR. By sensing capillary blood pressure and colloidal osmotic pressure, the filtered glomerular filtration fluid determines the amount of renal tubular reabsorption, maintaining ultrafiltration homeostasis.^47^ The results of the EMPA‐REG OUTCOME TM clinical study 30 showed that after the initial 4 weeks of treatment, the estimated GFR (eGFR) decreased for a short time in the englicine group. However, during the following long‐term treatment, eGFR remained stable in the englinide group and decreased progressively in the placebo group, indicating that englinide improves glomerular hyperfiltration and plays a long‐term renal protective role. In addition, Neal et al^34^ found that kaglitazine treatment reduced the risk of renal endpoint events by 40%.

## 5 CONCLUSION

At present, DKD is the main cause of ESRD, and its incidence remains high. Tubular feedback and TGF play a key role in maintaining the normal structure and function of renal tubules and glomeruli. Recently, it has been found that there are many common signalling pathways between TECs and GECs, and the crosstalk between them plays an important role in the occurrence and development of DKD. Improving the injury to TECs and GECs and maintaining normal crosstalk between them may become a new strategy for the prevention and treatment of DKD in the future. Further research efforts should be aimed at demonstrating that prevention of progression of the crosstalk between TECs and GECs is possible and leads to improved outcomes.

## CONFLICTS OF INTEREST

The authors have no conflicts of interest to declare.

## AUTHOR CONTRIBUTION

All authors contributed equally to manuscript writing.

## Data Availability

All data generated or analysed during this study are included in this article.
